# Dynamic hyperinflammatory response assessment using HIC scores in COVID-19: application to a large series of patients receiving anakinra

**DOI:** 10.3389/fimmu.2026.1722572

**Published:** 2026-05-22

**Authors:** Shirkhan Amikishiyev, Rabia Deniz, Mehmet Guven Gunver, Sarvan Aghamuradov, Nevzat Koca, Burak Ince, Murat Bektas, Aysenur Yilmaz, Yagmur Canturk, Gorkem Durak, Murat Kose, Mustafa Erelel, Arif Atahan Çağatay, Serap Simsek Yavuz, Sevgi Kalayoglu Besisik, Figen Esen, Ahmet Gül

**Affiliations:** 1 Department of Rheumatology, Istanbul Faculty of Medicine, Istanbul University, Istanbul, Türkiye; 2 Department of Internal Medicine, Faculty of Medicine and Health Sciences, Karabakh University, Khankendi, Azerbaijan; 3 Department of Internal Medicine, Istanbul Faculty of Medicine, Istanbul University, Istanbul, Türkiye; 4 Department of Biostatistics, Istanbul Faculty of Medicine, Istanbul University, Istanbul, Türkiye; 5 Department of Radiology, Istanbul Faculty of Medicine, Istanbul University, Istanbul, Türkiye; 6 Department of Pulmonology, Istanbul Medical Faculty, Istanbul University, Istanbul, Türkiye; 7 Department of Infectious Diseases and Clinical Microbiology, Istanbul Faculty of Medicine, Istanbul University, Istanbul, Türkiye; 8 Division of Hematology and Therapeutic Apheresis Unit, Istanbul Faculty of Medicine, Istanbul University, Istanbul, Türkiye; 9 Department of Anesthesiology, Istanbul Faculty of Medicine, Istanbul University, Istanbul, Türkiye

**Keywords:** anakinra, COVID-19, hemophagocytic lymphohistiocytosis, HIC score, hyperinflammation, macrophage activation syndrome, prognosis, treatment response prediction

## Abstract

**Introduction:**

Hyperinflammatory responses substantially contribute to morbidity and mortality in severe COVID-19 and share features with secondary hemophagocytic lymphohistiocytosis and macrophage activation syndrome. The Hyperinflammation in COVID-19 (HIC) criteria allow early diagnosis and may provide a framework for dynamic treatment monitoring.

**Methods:**

We retrospectively analyzed 218 hospitalized patients with hyperinflammation (HIC ≥35) who received anakinra at a tertiary referral center. The daily anakinra dose (100–800 mg/day, administered intravenously or subcutaneously) was adjusted according to clinical and laboratory parameters. The primary outcome was in-hospital mortality. Secondary outcomes included time to ≥50% CRP reduction, ICU admission, mechanical ventilation, and dynamic changes in ΔHIC and inflammatory biomarkers.

**Results:**

Overall mortality was 12.8%. Survivors achieved earlier CRP reduction than non-survivors (3.1 vs. 4.7 days) and showed a progressive decline in HIC, whereas non-survivors had persistently elevated or rising scores. Divergence in ΔHIC and other parameters, including neutrophil count, D-dimer, LDH, procalcitonin, and creatine kinase, emerged within 3–4 days. ROC analysis demonstrated that HIC on the day of anakinra initiation and at the final assessment discriminated survivors from non-survivors (AUC 0.75, p < 0.001; cut-offs 70.8 and 66.5, with high sensitivity but moderate specificity), whereas baseline and first-response-day HIC had limited predictive value (AUC approximately 0.50–0.55) .

**Discussion:**

These findings support the HIC score as both a diagnostic and dynamic monitoring tool during IL-1 blockade. Initiating anakinra when HIC is ≥35 but <70, and reassessing treatment response after 3–4 days using ΔHIC together with CRP kinetics, may help optimize outcomes.

## Introduction

Hyperinflammation is a well-recognized contributor to morbidity and mortality, characterized by the dysregulated innate immune responses and aberrant activation of inflammasomes, leading to excessive production of proinflammatory cytokines. A subset of patients develops hemophagocytic lymphohistiocytosis (HLH), a life-threatening syndrome characterized by fever, cytopenias, hyperferritinemia, coagulopathy, and multi-organ dysfunction (1, 2). HLH is classified into primary forms, caused by inherited defects of cytotoxic lymphocyte function, and secondary forms, which occur in association with infections, malignancy, or immune-mediated conditions (3). When secondary HLH arises in the context of systemic rheumatic or autoinflammatory diseases, it is often referred to as macrophage activation syndrome (4, 5). Importantly, among all triggers of secondary HLH/MAS, infections, particularly viral infections, account for the majority of cases. Epstein-Barr virus (EBV) remains the classical cause, but other viruses such as influenza, dengue, cytomegalovirus, parvovirus, and, more recently, SARS-CoV-2 (COVID-19) have also been implicated (6, 7).

The diagnosis of these hyperinflammatory conditions has been improved by advances in the understanding of their characteristics and the development of refined diagnostic criteria such as HLH-2004, HScore, MAS criteria, COVID-CS, COV-HI, and cHIS (1, 2, 8–11). However, the application of these criteria remains static and has not been validated for real-time monitoring of therapeutic responses. To address this issue, we recently developed the composite COVID-19-associated hyperinflammation criteria (HIC) score, which enables a dynamic assessment. A threshold ≥35 score provides an 85.3% sensitivity and 81.7% specificity (12). A schematic overview of the HIC domains, component variables, and scoring concept is provided in **Supplementary Figure 1**. Further evidence indicates that this composite score could also be a valuable tool for monitoring the response to tocilizumab treatment in patients with the HIC (13).

Anakinra, a recombinant interleukin-1 receptor antagonist (14) that blocks the activity of both IL-1α and IL-1β, has a remarkable record of safety with a short half-life of about 3–4 hours, has been used successfully in acquired forms of hemophagocytic lymphohistiocytosis such as MAS (15, 16), but there were conflicting reports about its use in COVID-19-associated hyperinflammation (**Supplementary Table 2**). Most of these studies used standard doses of anakinra for a limited period of time, and there was no tool to predict the treatment efficacy, which is critical in diseases with a rapidly progressive course and high mortality, such as COVID-19.

Building on our preliminary evaluation of predictors of response in a well-characterized cohort of COVID-19 patients with hyperinflammation treated with daily-adjusted doses of anakinra (17), the present study was designed to characterize clinical and laboratory predictors of anakinra response and to evaluate the performance of the HIC score as both a diagnostic tool and a dynamic monitoring instrument for treatment response and prognosis. Accordingly, the analysis was intentionally restricted to patients treated with anakinra in order to assess HIC and ΔHIC within a relatively defined therapeutic context of IL-1 blockade with individualized dose adjustment. Including the broader hospitalized COVID-19 population would have introduced substantial heterogeneity in immunomodulatory exposure, treatment timing, and evolving standards of care across different phases of the pandemic, thereby limiting the interpretability of trajectory-based response analyses.

## Patients and methods

### Patients

We conducted a retrospective cohort study and evaluated the clinical and laboratory parameters to define the prognostic factors in patients who received anakinra for the hyperinflammation of COVID-19. The records of hospitalized adult COVID-19 patients between March 2020 and May 2021 at Istanbul Faculty of Medicine, Istanbul University, Türkiye, which is a tertiary referral center heavily involved in the management of critical patients with COVID-19 during the pandemic, were used. The diagnosis of COVID-19 was confirmed by reverse-transcriptase Polymerase Chain Reaction (RT-PCR) positivity for SARS-CoV-2 on a nasopharyngeal swab and/or typical radiological findings at computed tomography (CT) in a symptomatic patient. All laboratory features and clinical findings were recorded using a standard form. The following clinical parameters were collected from the patients’ records: demographics, comorbidities, fever, and laboratory parameters at baseline (i.e., at the time of initial hospitalization), on the day anakinra treatment was started, on the day of the first response, and on the day patients were discharged from hospital or died. The laboratory data included neutrophils (normal range, 1.3-7.0 × 10^9^/L), lymphocytes (1.2-3.6 × 10^9^/L), monocytes (0-0.8 × 10^9^/L), platelets (160-390 × 10^9^/L), ferritin (30–400 ng/mL), D-dimer (0.0–550 ng/mL), CRP (0.0-5.0 mg/L), LDH (135–250 U/L), AST and ALT (5–45 U/L), procalcitonin (0-0.5 ng/mL), troponin (0–14 pg/mL), creatine kinase (30–220 U/L), fibrinogen (180–350 mg/dL), and creatinine (0.7-1.4 mg/dL). The patients received antiviral drugs according to the local guidelines, which changed throughout the pandemic. These included favipiravir, which was administered orally at a dose of 1600 mg twice daily on the first day and 600 mg twice daily from the second to the fifth or tenth day. Antibiotics were used when indicated. Enoxaparin was preferred as the anticoagulant therapy. Oxygen treatment was used in patients who had hypoxemia (SpO2 ≤94%) on room air.

The decision to initiate anakinra was based solely on expert clinical judgment at the time of treatment, before the formal establishment of the HIC criteria. Therefore, the diagnosis of a hyperinflammatory response relied on treating physicians interpreting clinical and laboratory features in real-time. The HIC framework was subsequently developed using the same dataset to standardize and quantify these features retrospectively for analysis.

Anakinra was administered to patients diagnosed with HIC. Twenty-two patients received anakinra after tocilizumab due to ongoing inflammatory parameters that did not respond to the initial treatment with tocilizumab. The decision to use glucocorticoids varied during the course of the COVID-19 pandemic. A total of 36 patients (16.5%) were given anakinra alongside standard care practices before September 2020. In the second period of the pandemic, patients with signs of hyperinflammatory response were treated first with glucocorticoids (dexamethasone 6 mg/day or the equivalent doses of methylprednisolone). Timing from symptom onset was not consistently available in the dataset; therefore, treatment timing was defined according to days from hospitalization. Anakinra was added to the treatment if there was no improvement in inflammatory parameters after a mean of 4.8 days of hospitalization-based follow-up. The dose of daily anakinra was determined according to the severity of clinical features and daily changes in laboratory parameters, varying from a subcutaneous dose of 100–300 mg/day to an intravenous dose of 400–800 mg/day. For patients with no comorbidities and who had severe lung parenchymal involvement, with the need for oxygen, and with high inflammatory parameters, especially CRP values above 100 mg/L, the anakinra dose decision changed between 400 and 800 mg/day. Lower anakinra doses were preferred in patients with milder symptoms than in this group. There was no pre-defined treatment period for anakinra administration, and it was given as long as the patient’s inflammatory parameters persisted by daily dose adjustments.

## Statistical analysis

### Study outcomes

As this was a retrospective observational study, outcomes were defined for analytic purposes prior to data analysis, although the HIC framework itself was derived from the same dataset. The primary outcome was in-hospital mortality and the predictive performance of HIC scores for mortality. Secondary outcomes included:

- Time to ≥ 50% reduction in C-reactive protein (CRP) from baseline (defining the “first response day”),
- Intensive care unit (ICU) admission,
- Need for invasive mechanical ventilation, and
- Dynamic changes in the HIC score (ΔHIC) and accompanying inflammatory biomarkers (CRP, ferritin, D-dimer, LDH, procalcitonin, fibrinogen, and creatine kinase) during the course of treatment.

### Statistical methods

Statistical analyses were performed using SPSS version 28 (IBM, Chicago, USA). Categorical variables are presented as counts and percentages, and comparisons between survivors and non-survivors were performed with the chi-square test. Continuous variables are summarized as mean ± standard deviation (SD). Normality was assessed by Shapiro–Wilk tests and Q–Q plots; non-normally distributed variables were log-transformed or analyzed with non-parametric tests as appropriate.

### Comparisons across time points

Laboratory parameters and HIC scores were evaluated at four key time points: baseline, day of anakinra initiation, first response day (defined as a ≥ 50% reduction in CRP), and the final assessment (discharge or death). Temporal changes were analyzed using repeated-measures ANCOVA or ANOVA, with age and anakinra dose included as covariates to adjust for potential confounding effects.

For the covariate analysis of drug exposure, the cumulative anakinra dose up to the first response day, corresponding to the time of ≥ 50% CRP reduction, was approximately 1400 mg, while the mean cumulative dose by the final assessment day (discharge or death) was approximately 3300 mg.

All comparative analyses between survivors and non-survivors were therefore interpreted within this dosing context.

For interval-based analyses, **Table 1** summarizes comparisons across the interval from the day of anakinra initiation to the first response day, whereas **Table 2** summarizes comparisons across the interval from the day of anakinra initiation to the final assessment (discharge or death).

**Table 1:** Longitudinal comparison of laboratory parameters between survivors and non-survivors across the interval from the day of anakinra initiation to the first response day (mean ± SD).

Laboratory parameter	Day of anakinra initiation		First response day		p-value
	Survivors	Non-survivors	Survivors	Non-survivors	
Neutrophil (×10^9^/L)	7.6 ± 0.3	8.7 ± 0.9	7.3 ± 0.2	9.1 ± 0.8	0.07
Lymphocyte (×10^9^/L)	1.1 ± 0.4	0.5 ± 1.1	1.2 ± 0.3	0.78 ± 1.0	0.86
Monocyte (×10^9^/L)	0.44 ± 0.02	0.43 ± 0.06	0.53 ± 0.02	0.53 ± 0.06	0.60
Platelet (×10^9^/L)	284 ± 9.6	285 ± 28.6	345.9 ± 10.6	300.7 ± 31.6	0.88
Ferritin (ng/mL)	1494.4 ± 140.6	1423.4 ± 410.7	1284 ± 105.4	1359 ± 307.8	0.48
D-dimer (ng/mL)	1742.9 ± 203.4	2571.4 ± 590.5	1503.9 ± 154.9	2932.9 ± 449.8	0.018
CRP (mg/L)	91.4 ± 5.5	141.8 ± 16	36 ± 2.4	52.2 ± 7.1	0.006
LDH (U/L)	404.4 ± 12	481.5 ± 31	354 ± 11.8	480 ± 33.9	0.003
ALT (U/L)	58.6 ± 4.7	56.3 ± 13.8	76.7 ± 7.3	61.1 ± 21	0.73
AST (U/L)	50 ± 3.3	55 ± 9.5	52 ± 5.7	45.5 ± 16.6	0.92
Procalcitonin (ng/mL)	0.26 ± 0.03	0.51 ± 0.1	0.15 ± 0.09	1.2 ± 0.27	0.005
Troponin (pg/mL)	67.1 ± 39.6	15.7 ± 114	65.5 ± 39	24.4 ± 112	0.63
Creatine kinase (U/L)	156.6 ± 20	303.7 ± 64	97.3 ± 22.6	354.3 ± 71.4	0.001
Fibrinogen (mg/dL)	613.6 ± 12	708 ± 37.8	549.7 ± 10.9	554.9 ± 33.4	0.05
Creatinine (mg/dL)	1.1 ± 0.05	1.07 ± 0.1	1.1 ± 0.08	1.5 ± 0.2	0.62

In the statistical analyses, age and anakinra dose were evaluated as covariates. For this interval-based analysis, the cumulative anakinra dose up to the first response day, defined as a ≥50% reduction in CRP, was approximately 1400 mg and was used as the representative dose value for covariate evaluation. Accordingly, comparisons between survivors and non-survivors reflect longitudinal differences across the interval from the day of anakinra initiation to the first response day. Sample size may vary across parameters because of missing measurements. Significant differences (p < 0.05) were observed for D-dimer, CRP, LDH, procalcitonin, creatine kinase, and fibrinogen between survivors and non-survivors.

CRP, C-reactive protein; LDH, lactate dehydrogenase; ALT, alanine aminotransferase; AST, aspartate aminotransferase.

**Table 2:** Longitudinal comparison of laboratory parameters between survivors and non-survivors across the interval from the day of anakinra initiation to the final assessment (discharge or death) (mean ± SD).

Laboratory parameter	Day of anakinra initiation		Final assessment (discharge or death)		p value
	Survivors	Non-survivors	Survivors	Non-survivors	
Neutrophil (×10^9^/L)	7.6 ± 0.3	8.6 ± 0.8	6.6 ± 0.3	10 ± 0.7	0.001
Lymphocyte (×10^9^/L)	0.74 ± 0.03	0.63 ± 0.07	1.3 ± 0.06	1.0 ± 0.1	0.03
Monocyte (×10^9^/L)	0.45 ± 0.02	0.43 ± 0.05	0.59 ± 0.03	0.64 ± 0.09	0.48
Platelet (×10^9^/L)	292.4 ± 10.7	254.6 ± 25.5	332.5 ± 11	229 ± 26.5	0.004
Ferritin (ng/mL)	1378 ± 115	1665.7 ± 284	780.9 ± 224.3	3024 ± 552.8	0.001
D-dimer (ng/mL)	1840.8 ± 236	2576.5 ± 559	984.3 ± 193.3	5342.7 ± 465.8	0.005
CRP (mg/L)	90.9 ± 6.0	143.1 ± 14.7	7.4 ± 4	155.8 ± 10	0.005
LDH (U/L)	402 ± 12	454.9 ± 28	266.9 ± 19.6	601.7 ± 46	0.005
ALT (U/L)	57.7 ± 5.4	51.1 ± 12.2	81.2 ± 7.9	124.7 ± 18.9	0.19
AST (U/L)	48.9 ± 3.4	54.5 ± 8	34.8 ± 15	227 ± 35.8	0.005
Procalcitonin (ng/mL)	0.24 ± 0.03	0.52 ± 0.08	0.21 ± 0.18	3.4 ± 0.45	0.005
Troponin (pg/mL)	75.5 ± 46.8	24.9 ± 114	27 ± 7	127 ± 17.4	0.51
Creatine kinase (U/L)	140.9 ± 18.7	173.5 ± 65.7	42.0 ± 15.7	424.8 ± 55.3	0.005
Fibrinogen (mg/dL)	605.8 ± 13.8	670.8 ± 34.8	385.8 ± 10.4	538.6 ± 26	0.005
Creatinine (mg/dL)	1.1 ± 0.06	1.2 ± 0.1	1.1 ± 0.08	1.2 ± 0.2	0.50

In the statistical analyses, age and anakinra dose were evaluated as covariates. For this interval-based analysis, the cumulative anakinra dose up to the final assessment day, defined as the day of discharge for survivors or the day of death for non-survivors, was approximately 3300 mg and was used as the representative cumulative exposure for covariate evaluation. Accordingly, comparisons between survivors and non-survivors reflect longitudinal differences across the interval from the day of anakinra initiation to the final assessment. Sample size may vary across parameters because of missing measurements. Significant differences (p < 0.05) were observed for neutrophil, lymphocyte, platelet, ferritin, D-dimer, CRP, LDH, AST, procalcitonin, creatine kinase, and fibrinogen levels between the two groups.

CRP, C-reactive protein; LDH, lactate dehydrogenase; ALT, alanine aminotransferase; AST, aspartate aminotransferase.

### Definition of ΔHIC

ΔHIC was defined as the difference between follow-up and index HIC values (*ΔHIC = HIC_{follow-up} – HIC_{index}*). Negative ΔHIC indicated improvement, while positive values reflected worsening of hyperinflammation.

### Discrimination analysis

Discrimination for mortality was assessed using receiver operating characteristic (ROC) curves for HIC at four time points (baseline, day of anakinra initiation, first response day, and final assessment). Areas under the curve (AUCs) with 95% confidence intervals (CIs) were estimated, and p-values for the null hypothesis AUC = 0.5 were calculated. Optimal cut-offs were determined by Youden’s J statistic, with corresponding sensitivity and specificity reported.

### Statistical significance

A two-sided α level of 0.05 was considered statistically significant. Given the retrospective and hypothesis-generating design of the study, p-values were not adjusted for multiple comparisons and should be interpreted in an exploratory context.

## Results

Out of 1,080 hospitalized patients with a confirmed diagnosis of SARS-CoV-2, 218 patients (151 men and 67 women, with a mean age of 60.0 ± 0.9 years, ranging from 24 to 96 years) who received anakinra were identified. Baseline demographic and clinical characteristics of survivors and non-survivors are presented in **Table 3**. Non-survivors were older and had a higher prevalence of selected cardiovascular-metabolic comorbidities, particularly hypertension, diabetes mellitus, coronary artery disease, congestive heart failure, and chronic kidney disease. The most frequent comorbidities overall were hypertension and diabetes mellitus. Hospital length of stay was also longer in non-survivors than in survivors.

**Table 3:** Baseline demographic and clinical characteristics of survivors and non-survivors treated with anakinra.

Variable	Survivors (n = 190)	Non-survivors (n = 28)	p value
Age, years	58.9 ± 13.9	66.2 ± 13.9	0.013
Male sex, n (%)	135 (71.1)	16 (57.1)	0.136
RT-PCR positivity, n (%)	166 (87.4)	22 (78.6)	0.207
Any comorbidity, n (%)	132 (69.5)	23 (82.1)	0.167
>2 comorbidities, n (%)	95 (50.0)	18 (64.3)	0.158
Hypertension, n (%)	75 (39.5)	20 (71.4)	0.001
Diabetes mellitus, n (%)	42 (22.1)	11 (39.3)	0.048
Coronary artery disease, n (%)	17 (8.9)	8 (28.6)	0.002
Chronic pulmonary disease, n (%)	10 (5.3)	0 (0.0)	0.368
Congestive heart failure, n (%)	3 (1.6)	4 (14.3)	0.006
Chronic kidney disease, n (%)	9 (4.7)	5 (17.9)	0.008
Renal transplantation, n (%)	7 (3.7)	0 (0.0)	0.599
Cerebrovascular disease, n (%)	7 (3.7)	1 (3.6)	1.000
Dementia, n (%)	6 (3.2)	1 (3.6)	1.000
Peripheral artery disease, n (%)	4 (2.1)	0 (0.0)	1.000
Systemic lupus erythematosus, n (%)	4 (2.1)	0 (0.0)	1.000
Rheumatoid arthritis, n (%)	1 (0.5)	0 (0.0)	1.000
Familial Mediterranean fever, n (%)	4 (2.1)	0 (0.0)	1.000
Solid-organ malignancy, n (%)	13 (6.8)	1 (3.6)	1.000
Hematologic malignancy, n (%)	4 (2.1)	1 (3.6)	0.501
Hospital length of stay, days, median (IQR)	13.5 (11.0-20.0)	21.5 (13.0-25.0)	0.004

Data are presented as mean ± SD or n (%), as appropriate.

Comparisons between survivors and non-survivors were performed using the chi-square test or Fisher’s exact test for categorical variables and the independent-samples t-test or Mann-Whitney U test for continuous variables, as appropriate.

Hospital length of stay is presented as median (IQR).

Comorbidities are presented as baseline between-group associations and should not be interpreted as independent causal determinants of mortality.

RT-PCR, reverse-transcriptase polymerase chain reaction.

Anakinra treatment was started at a mean of 4.8 days (range 1-27) after hospitalization, which was the consistently available timing metric in this retrospective dataset. Of the patients with fever and/or hypoxemia and hyperinflammation, 83.5% (n = 182) received glucocorticoid treatment (79.5% methylprednisolone and 5% dexamethasone). Six patients received a single 250 mg intravenous dose of methylprednisolone. Thirty-six (16.5%) patients, who followed before September 2020, received anakinra without prior use of glucocorticoid treatment.

In the earliest period of the pandemic, the algorithm for hospitalization was different, and most symptomatic patients were followed at the hospital without taking into account oxygen support. Consequently, 21 patients (9.6%) did not require supplemental oxygen. Overall, 152 (69.7%) patients were followed in the ward, 66 (30.3%) followed at the ICU, and 40 (18.3%) of them were intubated. Of the patients who started anakinra treatment after receiving tocilizumab, 9 were observed in the service, 13 in the ICU, 10 were intubated, and 6 died during this period.

The primary outcome, in-hospital mortality, occurred in 28 patients (12.8%). Among non-survivors, 16 were male and 12 were female, with a mean age of 66.2 ± 2.6 years (range, 34–88). Twenty-two (78.6%) had RT-PCR-confirmed SARS-CoV-2 infection at baseline.

Four patients with marked acute-phase hyperinflammation developed sudden cardiopulmonary arrest, whereas the remaining non-survivors succumbed primarily to sepsis secondary to bacterial infection, and 13 (46%) developed multi-organ failure during hospitalization. All non-survivors exhibited markedly elevated troponin levels, suggesting cardiac involvement in the terminal phase.

The secondary outcomes included dynamic changes in HIC by days 3-4, time to achieve ≥ 50% CRP reduction, ICU admission, need for invasive mechanical ventilation, and serial changes in inflammatory biomarkers throughout follow-up. The mean time to achieve a ≥50% reduction in CRP was 3.1 days in survivors and 4.7 days in non-survivors. ICU admission was required in 66 patients (30.3%), and 40 (18.3%) required mechanical ventilation. Survivors demonstrated a progressive decline in HIC, CRP, D-dimer, and LDH, whereas non-survivors exhibited persistently elevated or increasing values, consistent with uncontrolled hyperinflammation and poor therapeutic response.

Several comparative analyses were performed on survivors and non-survivors following exposure to anakinra, using inflammatory parameters at baseline, on the day of anakinra initiation, on the first response day (defined as a 50% reduction in CRP), and at the final assessment (discharge or death).

### Baseline evaluations at hospitalization

At baseline, univariate analyses revealed significantly higher CRP levels in non-survivors than in survivors (p = 0.05), although other parameters were similar (**Supplementary Table 1**).

### Anakinra initiation

On the day of anakinra initiation, CRP and procalcitonin values were significantly lower in the surviving patients (p = 0.002 and p = 0.003, respectively, **Supplementary Table 1**).

### First response day

On the first response day, neutrophil count (p = 0.02), D-dimer (p = 0.02), LDH (p = 0.001), CRP (p = 0.03), procalcitonin (p = 0.005), and creatine kinase levels (p = 0.002) were significantly lower in the surviving patients (**Supplementary Table 1**).

### Final assessment

In the comparative analysis of the parameters on the day of discharge or death, neutrophil and lymphocyte counts, and concentrations of ferritin, D-dimer, CRP, LDH, ALT, AST, procalcitonin, troponin, creatine kinase, and fibrinogen were significantly higher in the non-survivors (**Supplementary Table 1**).

The mean time to 50% CRP reduction was 3.1 days in survivors and 4.7 days in non-survivors.

Comparative measurements between the day of anakinra initiation and the first response day for the survivors and non-survivors are given in **Table 1**, which shows significant differences in D-dimer (p = 0.018), CRP (p = 0.006), LDH (p = 0.003), procalcitonin (p = 0.005), creatine kinase (p = 0.001), and fibrinogen levels (p = 0.05).

Comparisons between the day of anakinra initiation and the final assessment (discharge or death) for the survivors and non-survivors are given in **Table 2**. In this analysis, neutrophil count (p=0.001), lymphocyte count (p = 0.03), ferritin (p = 0.001), D-dimer (p = 0.005), CRP (p = 0.005), LDH (p = 0.005), AST (p = 0.005), procalcitonin (p = 0.005), creatine kinase (p = 0.005), and fibrinogen levels (p = 0.005) were significantly different between the two groups of patients (**Table 2**).

**Figure 1:**
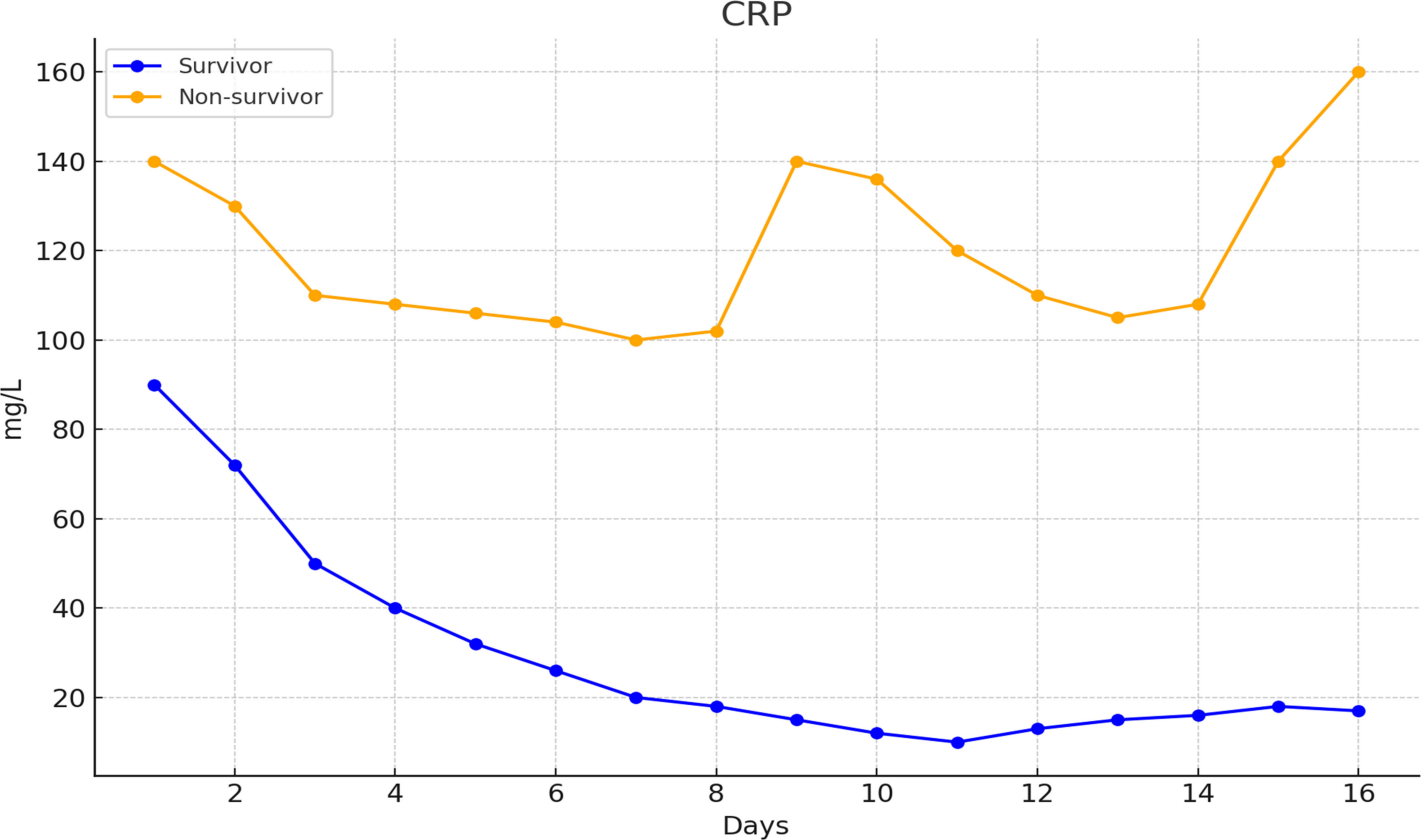
CRP dynamics in survivors and non-survivors.

We then used CRP concentration as the main parameter for monitoring the response to treatment. We evaluated CRP throughout the dynamic course of the two patient groups in association with the anakinra dose. **Figure 1** shows the mean CRP value changes in the survivors and non-survivors. The non-survivors had higher CRP values and did not experience a sustained decrease in CRP levels during treatment with steroids and anakinra. In **Supplementary Figure 2**, the CRP concentrations were categorized according to baseline CRP levels: less than 50 mg/L, between 50 and 100 mg/L, between 100 and 150 mg/L, and greater than 150 mg/L. This was done for both survivors and non-survivors. Despite having different initial values, the surviving patients showed a similar trend of decreases in CRP concentrations. However, no such response pattern was observed in the non-survivors.

The CRP concentration changes in association with the initial daily anakinra doses of 400 mg, 600 mg, and 800 mg in the survivors and non-survivors are given in **Supplementary Figure 3**. The non-survivors had higher CRP values at the beginning of anakinra treatment; they experienced a more prominent decrease if they received 800 mg of anakinra initially. The non-survivors also had more prominent lymphopenia and neutrophilia when the mean lymphocyte and neutrophil counts were assessed (**Supplementary Figure 4**).

In the serial supplementary plots, days refer to days from anakinra initiation; the number of evaluable patients decreased over time because of discharge, death, and occasional missing measurements.

**Figure 2:**
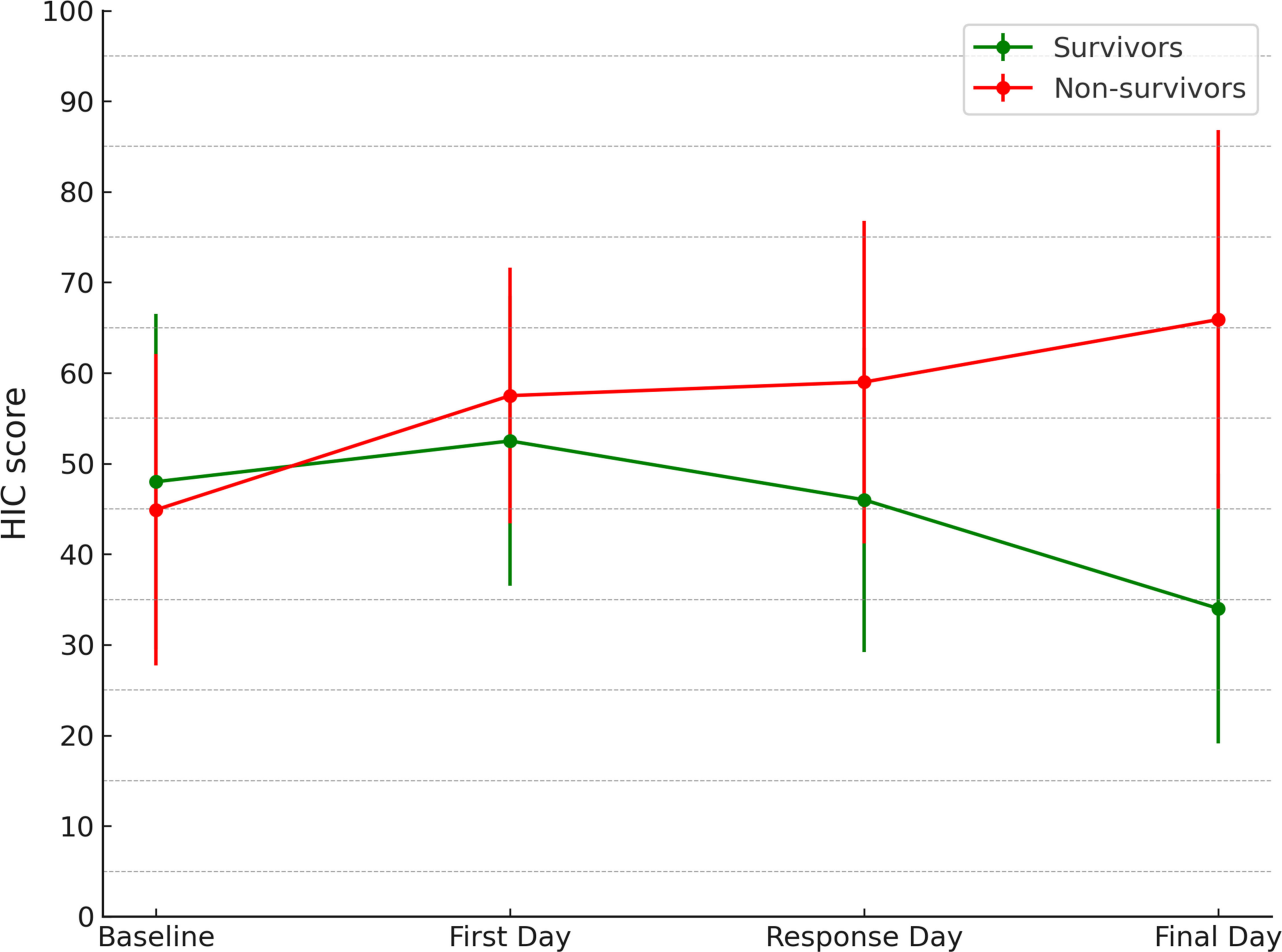
HIC/ΔHIC dynamics in survivors and non-survivors with COVID-19. ΔHIC dynamics in survivors and non-survivors. Negative ΔHIC denotes improvement (follow-up HIC below index), while positive ΔHIC indicates worsening. The dashed line marks the diagnostic threshold (HIC = 35).

Dynamic evaluation of ΔHIC showed an early divergence between groups. Survivors typically had negative ΔHIC values (improvement) by day 3-4, whereas non-survivors demonstrated positive ΔHIC (progression of hyperinflammation) (**Figure 2**). At baseline, scores were comparable in both groups (48.0 ± 18.5 vs. 44.9 ± 17.2). On the day of anakinra initiation, both groups exceeded the diagnostic threshold, with survivors at 52.5 ± 16.0 and non-survivors at 57.5 ± 14.1. By the first response day, survivors showed a decline to 46.0 ± 16.8, whereas non-survivors worsened further to 59.0 ± 17.8 despite an early decrease in CRP values. This divergence became even more pronounced by the final assessment. The survivors had a decrease in mean HIC scores to 34.0 ± 14.9, while the mean scores in non-survivors remained persistently elevated (65.9 ± 20.9).

Consistent with the secondary outcomes, ROC analysis demonstrated that HIC measured on the day of anakinra initiation and at the final assessment significantly discriminated survivors from non-survivors (AUC 0.746; 95% CI 0.627-0.864; p<0.0001 and AUC 0.746; 95% CI 0.619-0.873; p = 0.0001, respectively). The optimal cut-off values were 70.8 (sensitivity 0.88; specificity 0.55) for the day of anakinra initiation and 66.5 (sensitivity 0.83; specificity 0.61) for the final assessment. Baseline and first-response-day HIC showed limited discrimination (AUC 0.497; 95% CI 0.385-0.608; p = 0.955 and AUC 0.546; 95% CI 0.404-0.689; p = 0.525). Full metrics are provided in **Table 4**, and ROC curves are shown in **Supplementary Figure 5**.

**Table 4:** Receiver operating characteristic (ROC) analysis of HIC scores for the prediction of in-hospital mortality in anakinra-treated patients.

Timepoint	AUC	95% CI	p-value	Cut-off	Sensitivity	Specificity
Baseline	0.497	0.385-0.608	0.9550	17.9	1.00	0.12
Day of anakinra initiation	0.746	0.627-0.864	<0.0001	70.8	0.88	0.55
First response day	0.546	0.404-0.689	0.5252	95.6	0.37	0.76
Final assessment	0.746	0.619-0.873	0.0001	66.5	0.83	0.61

Statistically significant results (p < 0.05, bold) were observed for the HIC scores on the day of anakinra initiation and at the final assessment.

ROC, Receiver Operating Characteristic; AUC, Area Under the Curve; CI, Confidence Interval.

## Discussion

This study demonstrated that the HIC score, developed to diagnose and monitor hyperinflammation associated with SARS-CoV-2 infection, is a reliable diagnostic method and a dynamic monitoring tool for evaluating treatment responses. Survivors of COVID-19-associated hyperinflammation consistently showed an early and sustained reduction in composite HIC scores and individual inflammatory parameters, whereas non-survivors did not, despite similar baseline HIC values.

Our primary outcome, in-hospital mortality, was 12.8% and served as the central benchmark for evaluating treatment efficacy. Secondary outcomes contextualized these findings: ICU admission occurred in 66 patients (30.3%), mechanical ventilation was required in 40 (18.3%), and survivors achieved a ≥50% CRP reduction earlier than non-survivors (3.1 vs. 4.7 days).

ROC analysis further reinforced these observations and outlined a practical “hypothetical treatment window” for anti-inflammatory therapy. At the time of anakinra initiation and at the final assessment, the HIC score showed good discrimination for in-hospital mortality (AUC 0.75; 95% CI 0.63-0.86; p < 0.001). The optimal cut-offs of 70.8 and 66.5 provided high sensitivity (0.88 and 0.83) with moderate specificity (0.55 and 0.61). Importantly, these data suggest that once the HIC score approaches ~70, outcomes become much less reversible, whereas treatment initiated within the HIC ≥ 35 but < 70 range is associated with better trajectories. This pattern of limited discrimination at baseline but strong post-treatment separation indicates that HIC captures the inflammatory trajectory rather than static severity. From a clinical perspective, identifying patients at risk of entering a non-recoverable hyperinflammatory phase is important. This can be achieved by setting an early-day threshold around 70, which may indicate the need for prompt intensification or switching of therapy. Although these findings are retrospective, they support the early initiation and rapid reassessment required to prevent progression into the high-risk HIC zone (zone 70).

The relatively low discriminatory performance of HIC on the first response day likely reflects the nature of this time point itself. Unlike treatment initiation or final assessment, the first response day was defined by an early CRP-based biochemical milestone rather than by a fixed and stable clinical state. This intermediate stage may therefore have compressed between-patient variability and reduced separation between survivors and non-survivors. In contrast, HIC at the day of anakinra initiation reflects the initial inflammatory burden, whereas HIC at the final assessment reflects the more fully evolved divergence of clinical trajectories.

This response-adaptive approach aligns with prior evidence that biomarker-guided early anakinra administration can improve outcomes, as demonstrated in the SAVE-MORE trial, where suPAR ≥6 ng/mL was used to identify candidates for early treatment (18). Our study extends this principle by applying a composite, widely available laboratory score (HIC) rather than a single biomarker. Importantly, all HIC components are derived from routine laboratory tests, making implementation feasible in most hospital settings. Furthermore, in patients with IL-6 blockade, where CRP levels are artificially suppressed (13), ΔHIC retained its predictive power. A further point relevant to generalizability is that, although the principle of repeated composite-score monitoring may extend to other virus-associated hyperinflammatory syndromes, direct extrapolation of HIC to all secondary HLH/MAS settings should be made cautiously. Some HIC components may be confounded by the underlying disease itself, for example, baseline cytopenias in systemic lupus erythematosus, thereby affecting score interpretation independently of treatment response.

Several scoring systems, including HLH-2004, HScore, MAS criteria, COVID-CS, COV-HI, and cHIS, have been used for case definition, but they are inherently static and not designed for repeated application during anti-inflammatory therapy (1, 2, 8–11). The ability of HIC to discriminate between survivors and non-survivors as early as the first reassessment supports its value as a dynamic monitoring tool in COVID-19 hyperinflammation.

Our cohort adds to the expanding yet heterogeneous body of evidence on the use of anakinra, representing the largest reported COVID-19 cohort to date. One study demonstrated decreased mortality (35.6% in the control group and 13.9% in the anakinra group) among patients with a high acute-phase response who required oxygen (19). Several observational studies reported improved outcomes with anakinra, particularly when combined with glucocorticoids in patients with evidence of hyperinflammation (20–24). However, four studies reported no significant differences in outcomes between the anakinra and control groups (**Supplementary Table 2**) (25–28). In contrast, glucocorticoids were shown to reduce adverse outcomes in COVID-19 in the RECOVERY trial (29), and the majority of the anakinra studies were conducted in conjunction with glucocorticoid therapy. A recent meta-analysis likewise did not demonstrate a consistent mortality reduction (30), underscoring the influence of patient selection, timing of intervention, and dosing strategies. Against this variable backdrop, our study’s response-adaptive, dose-adjusted approach, coupled with dynamic monitoring via ΔHIC and CRP, offers a pragmatic framework that may help standardize decision-making across settings.

Early initiation and adequate dosing also emerged as critical factors. In our series, most patients (83.5%) received anakinra only after an insufficient response to glucocorticoids, with a mean delay of 4.8 days. By this stage, HIC scores were already well above the diagnostic threshold. Survivors demonstrated a progressive decline, whereas non-survivors maintained persistently high scores. In some refractory patients, high-dose intravenous anakinra was effective in achieving biochemical improvement; however, delayed initiation of treatment likely limited the survival benefit. These findings suggest that earlier initiation (at HIC ≥35) and response-guided dose escalation may optimize outcomes. Conversely, persistently high HIC and CRP after 3–4 days should prompt escalation.

In patients who died, HIC scores increased rapidly between baseline and day of anakinra initiation, reflecting uncontrolled hyperinflammation. The subgroup that received anakinra after tocilizumab illustrates this severity; notably, 16 of 22 patients responded favorably despite prior refractory disease. In this context, CRP interpretation was limited due to IL-6 blockade, but ΔHIC maintained prognostic value, underscoring its robustness as a dynamic monitoring tool.

**Figure 3:**
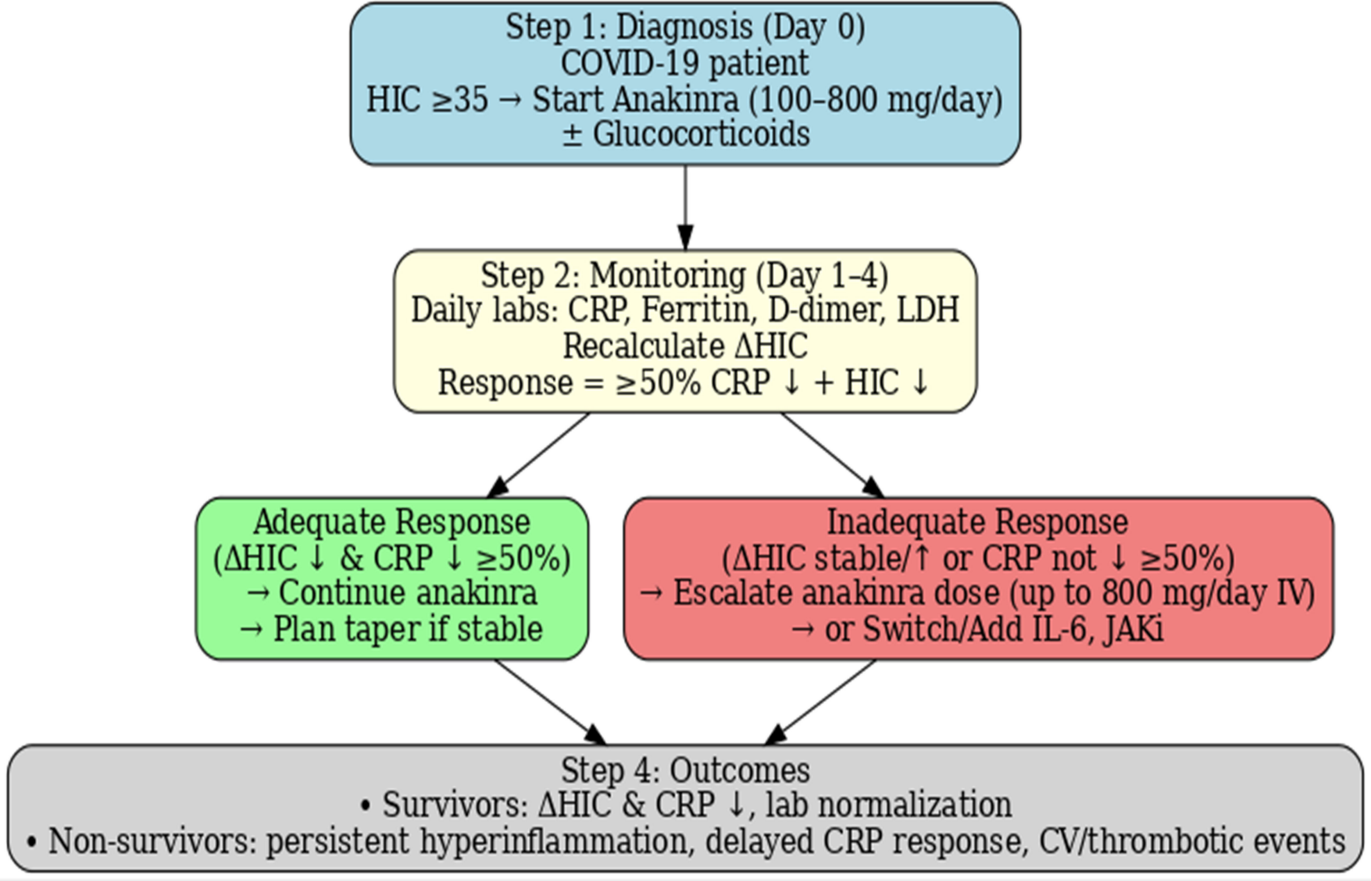
ΔHIC-guided management algorithm. HIC-guided management algorithm for COVID-19 hyperinflammation. Patients with HIC ≥35 are candidates for IL-1 blockade (anakinra ± glucocorticoids). Treatment response should be reassessed after 3–4 days using ΔHIC and CRP. A ≥50% CRP reduction with concurrent HIC improvement indicates a favorable response and continuation of therapy. Lack of improvement warrants dose escalation or switching to alternative cytokine-targeted therapies (e.g., IL-6 blockade, JAK inhibitors). The principle of dynamic score monitoring may also apply to other virus-associated hyperinflammatory syndromes (e.g., EBV, influenza, dengue, CMV) that can produce ARDS-like manifestations.

Collectively, these results underscore the value of a response-adaptive management strategy for COVID-19 hyperinflammation. To illustrate this principle, we provide a visual algorithm (**Figure 3**) based on HIC and CRP-guided monitoring, emphasizing early reassessment (day 3-4) as the critical decision point for therapy adjustment.

The main limitations of this study are its retrospective design, the absence of a control group, and the heterogeneity introduced by evolving treatment strategies across pandemic phases. Nevertheless, this study represents one of the largest single-center cohorts of anakinra-treated patients and is strengthened by individualized dose adjustment and systematic monitoring of dynamic inflammatory responses.

Although the HIC framework may have broader prognostic applicability, this analysis was intentionally restricted to anakinra-treated patients in order to evaluate dynamic score behavior under a defined intervention. Prospective controlled studies should determine whether the same monitoring principles apply in non-anakinra cohorts and across other immunomodulatory strategies.

In conclusion, the HIC score serves a dual role: as a diagnostic threshold for COVID-19-associated hyperinflammation and as a dynamic response-monitoring tool during IL-1 blockade. ROC results (AUC ≈ 0.75 after treatment initiation) support its prognostic utility, particularly when evaluated together with CRP kinetics. Importantly, our findings indicate a therapeutic “hypothetical treatment window” between HIC ≥35 and <70, within which anti-inflammatory interventions such as anakinra appear most effective. Once the HIC score approaches or exceeds ~70, outcomes become poorly reversible, suggesting that earlier initiation and reassessment may prevent transition into this high-risk range.

Therefore, initiating anakinra when HIC is ≥35, reassessing at 3–4 days using ΔHIC plus CRP, and adapting therapy accordingly by escalating or switching if improvement is lacking, may help optimize outcomes. Prospective, controlled trials should explicitly evaluate this ΔHIC/CRP-guided, threshold-based approach in COVID-19 and other virus-associated MAS/secondary HLH syndromes.

## Data Availability

The anonymized data supporting the conclusions of this article will be made available by the authors upon reasonable request, without undue reservation.

## Supplementary material

Supplementary Figure 1 Domains and component variables of the Hyperinflammation in COVID-19 (HIC) score. Schematic overview of the 12-item HIC framework, including the component variables, composite scoring principle, and diagnostic threshold (HIC ≥35) previously shown to identify COVID-19-associated hyperinflammation. In the present study, this framework was applied serially at baseline, the day of anakinra initiation, the first response day, and the final assessment (discharge or death) to evaluate dynamic changes in hyperinflammatory burden. Direct extrapolation to all secondary HLH/MAS settings should be made cautiously, since some component variables may be influenced by the underlying disease process itself.

Supplementary Figure 2 Levels of CRP changes (<50, 50-100, 100-150, >150 mg/L) in survivors and non-survivors. Days on the x-axis indicate days from anakinra initiation. Curves are displayed up to day 16 to facilitate comparison of the early treatment trajectory. The number of evaluable patients decreased over time because of discharge, death, and occasional missing measurements; therefore, later time points reflect progressively smaller denominators.

Supplementary Figure 3 CRP changes in survivors and non-survivors according to the anakinra doses (400, 600, and 800 mg doses/day). Days on the x-axis indicate days from anakinra initiation. Curves are displayed up to day 16 to facilitate comparison of the early treatment trajectory. The number of evaluable patients decreased over time because of discharge, death, and occasional missing measurements; therefore, later time points reflect progressively smaller denominators.

Supplementary Figure 4 Mean lymphocyte and neutrophil count changes in survivors and non-survivors. Days on the x-axis indicate days from anakinra initiation. Curves are displayed up to day 16 to facilitate comparison of the early treatment trajectory. The number of evaluable patients decreased over time because of discharge, death, and occasional missing measurements; therefore, later time points reflect progressively smaller denominators.

Supplementary Figure 5 ROC curves of HIC scores for mortality prediction. ROC curve analysis for HIC scores at different time points (baseline, day of anakinra initiation, first response day defined by ≥50% CRP reduction, and final assessment). AUC values with 95% confidence intervals (CI), *p*-values for the null hypothesis (AUC = 0.5), optimal cut-off values (Youden index), sensitivity, and specificity are shown. Significant discriminatory ability was observed on the day of anakinra initiation and final assessment, but not at baseline or first response day.

Supplementary Table 1 Inflammatory parameters (mean ± SD) at baseline, day of anakinra initiation, first response day, and final assessment (discharge or death). **Supplementary Table 1** presents absolute parameter values (mean ± SD) at each predefined time point. Sample size may vary across parameters and time points because of missing measurements and differences in follow-up duration. Significant differences (p < 0.05) were observed mainly at the response and final assessment stages, highlighting delayed divergence in inflammatory trajectories between survivors and non-survivors. CRP, C-reactive protein; LDH, lactate dehydrogenase; ALT, alanine aminotransferase; AST, aspartate aminotransferase.

Supplementary Table 2 Summary of inclusion criteria, glucocorticoid (GC) use, anakinra regimens, main outcomes, and mortality. A, anakinra; C, control; CRP, C-reactive protein; FiO_2_, fraction of inspired oxygen; PaO_2_, partial pressure of oxygen; suPAR, soluble urokinase plasminogen activator receptor; LDH, lactate dehydrogenase; RR, respiratory rate; IMV, invasive mechanical ventilation; WHO-CPS, World Health Organization Clinical Progression Scale. *p < 0.05* indicates statistical significance where reported.
