# Supplementary figures and images for: Dynamic hyperinflammatory response assessment using HIC scores in COVID-19: application to a large series of patients receiving anakinra
Source: Front Immunol. 2026 May 22;17:1722572. doi: 10.3389/fimmu.2026.1722572 (PMC13236672; doi:10.3389/fimmu.2026.1722572)

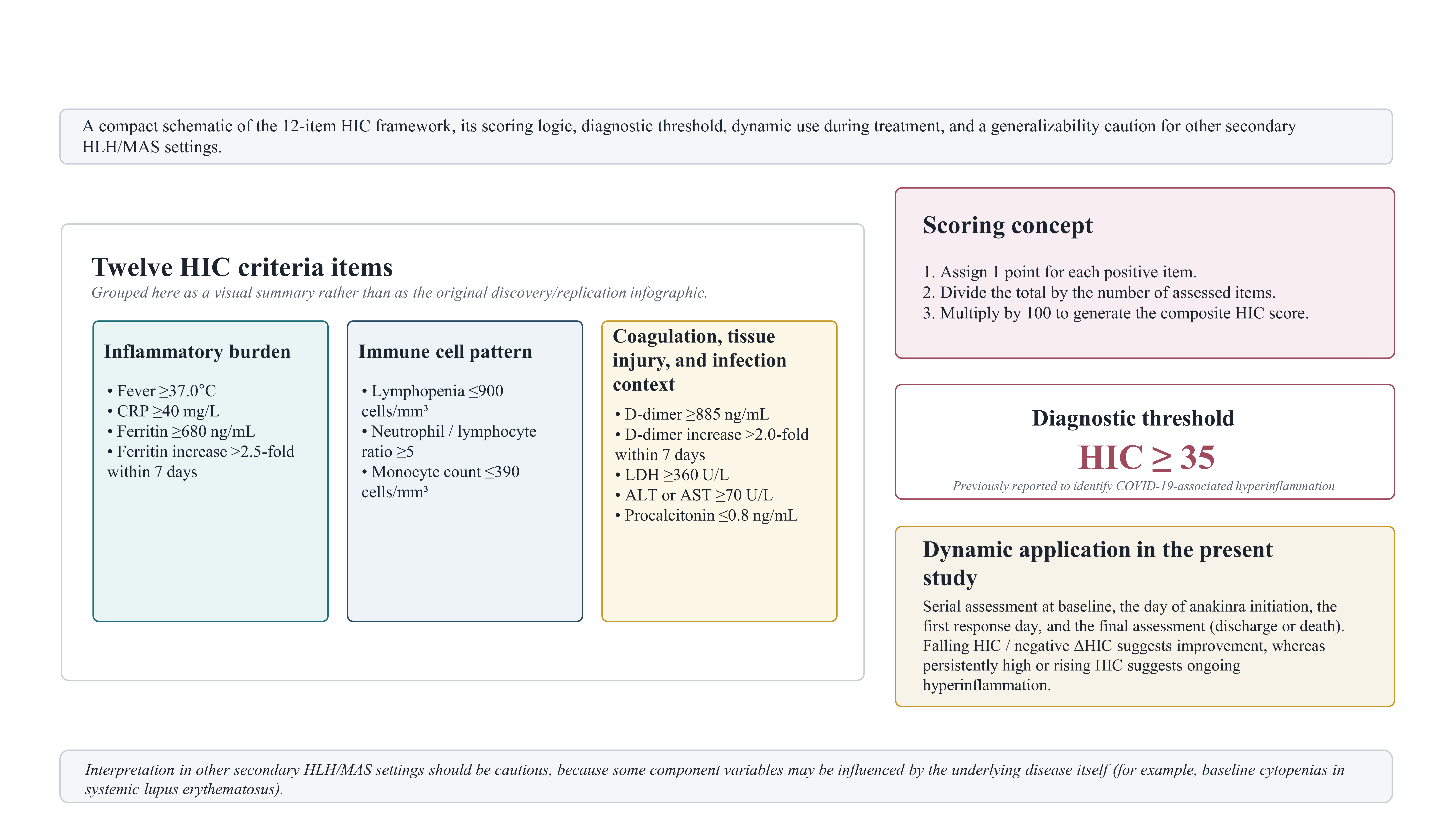

Supplement: Supplementary Figure 1 — Domains and component variables of the Hyperinflammation in COVID-19 (HIC) score. Schematic overview of the 12-item HIC framework, including the component variables, composite scoring principle, and diagnostic threshold (HIC ≥35) previously shown to identify COVID-19-associated hyperinflammation. In the present study, this framework was applied serially at baseline, the day of anakinra initiation, the first response day, and the final assessment (discharge or death) to evaluate dynamic changes in hyperinflammatory burden. Direct extrapolation to all secondary HLH/MAS settings should be made cautiously, since some component variables may be influenced by the underlying disease process itself. [file Image1.tif]

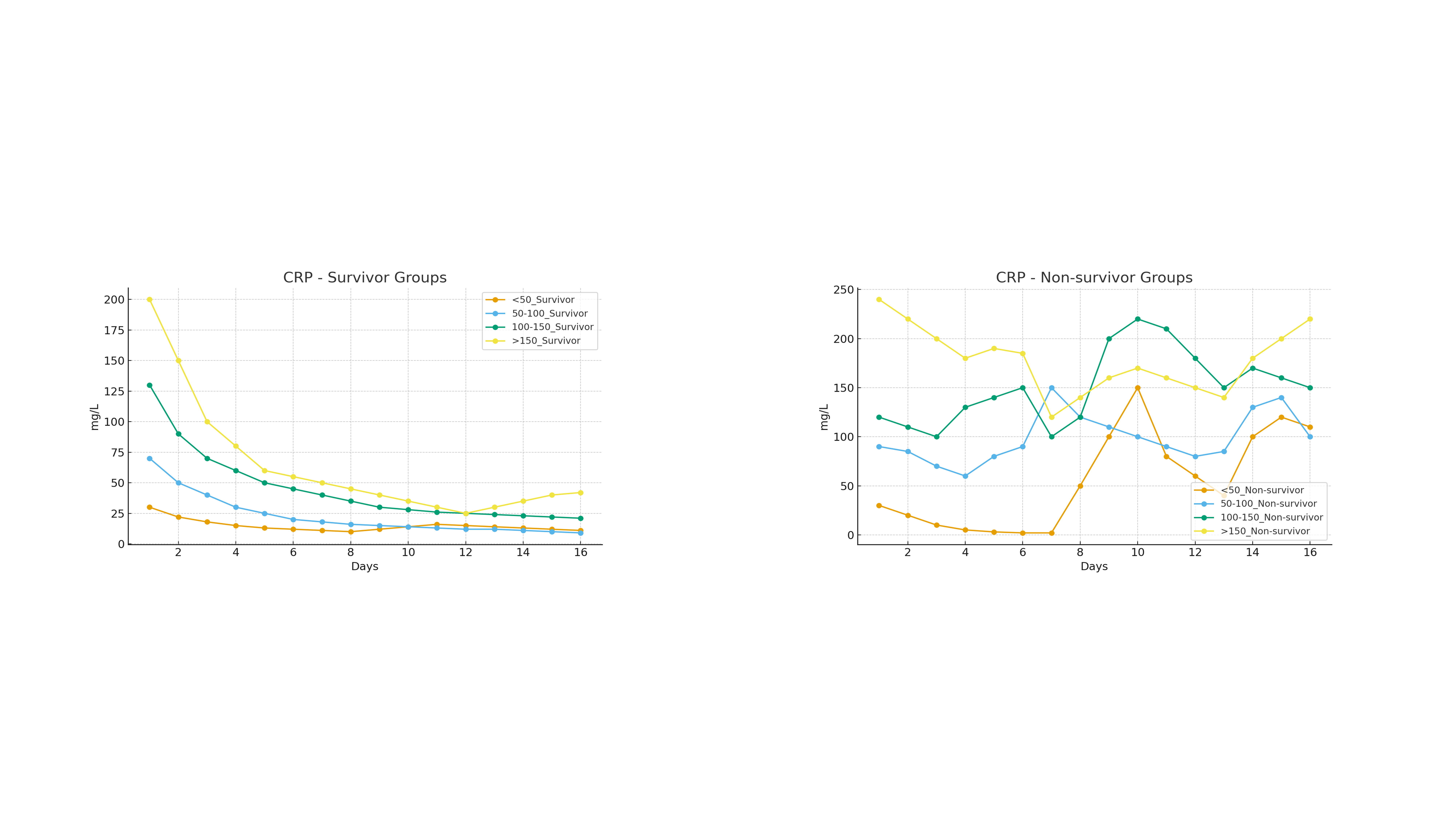

Supplement: Supplementary Figure 2 — Levels of CRP changes (<50, 50-100, 100-150, >150 mg/L) in survivors and non-survivors. Days on the x-axis indicate days from anakinra initiation. Curves are displayed up to day 16 to facilitate comparison of the early treatment trajectory. The number of evaluable patients decreased over time because of discharge, death, and occasional missing measurements; therefore, later time points reflect progressively smaller denominators. [file Image2.tif]

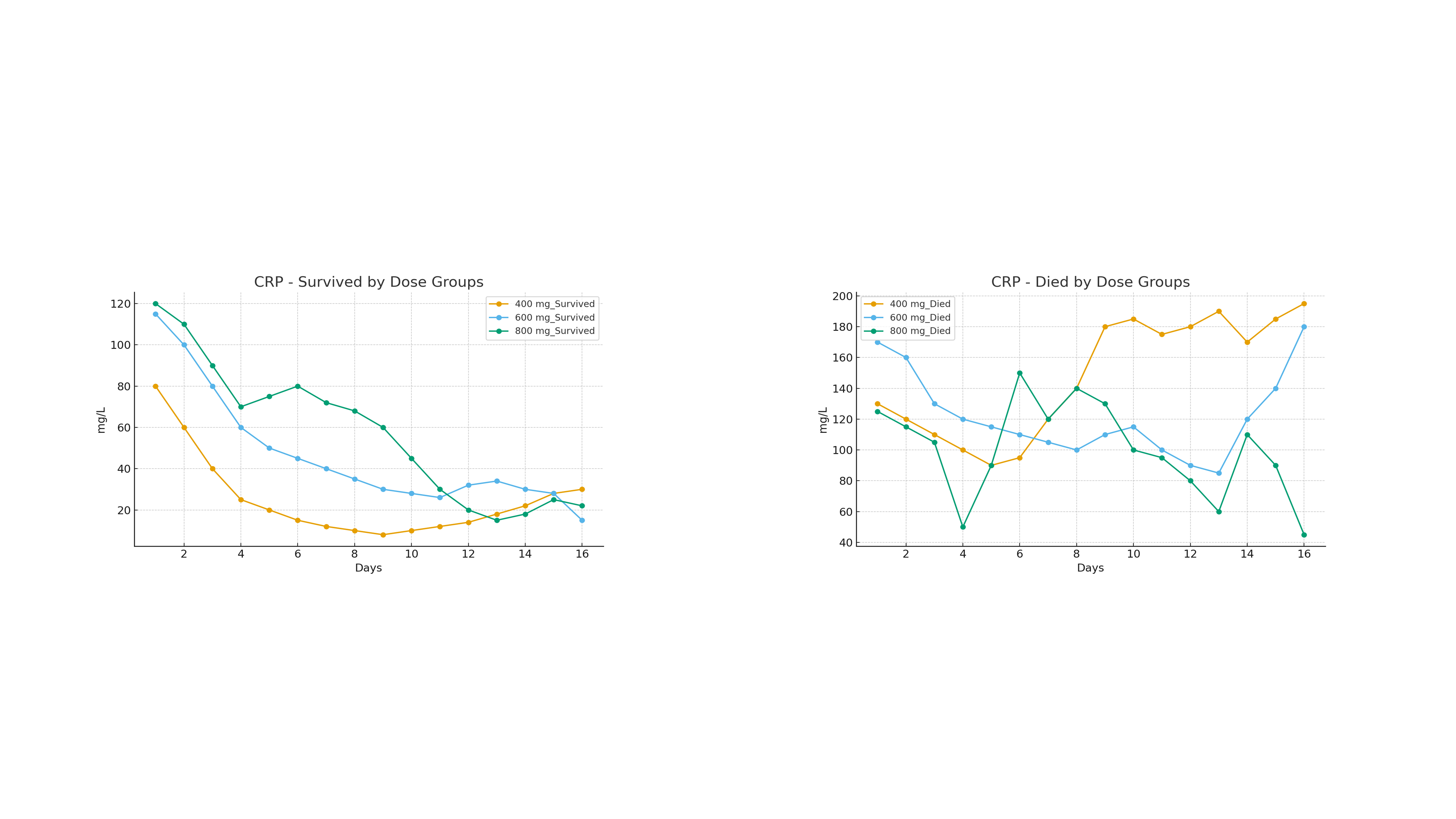

Supplement: Supplementary Figure 3 — CRP changes in survivors and non-survivors according to the anakinra doses (400, 600, and 800 mg doses/day). Days on the x-axis indicate days from anakinra initiation. Curves are displayed up to day 16 to facilitate comparison of the early treatment trajectory. The number of evaluable patients decreased over time because of discharge, death, and occasional missing measurements; therefore, later time points reflect progressively smaller denominators. [file Image3.tif]

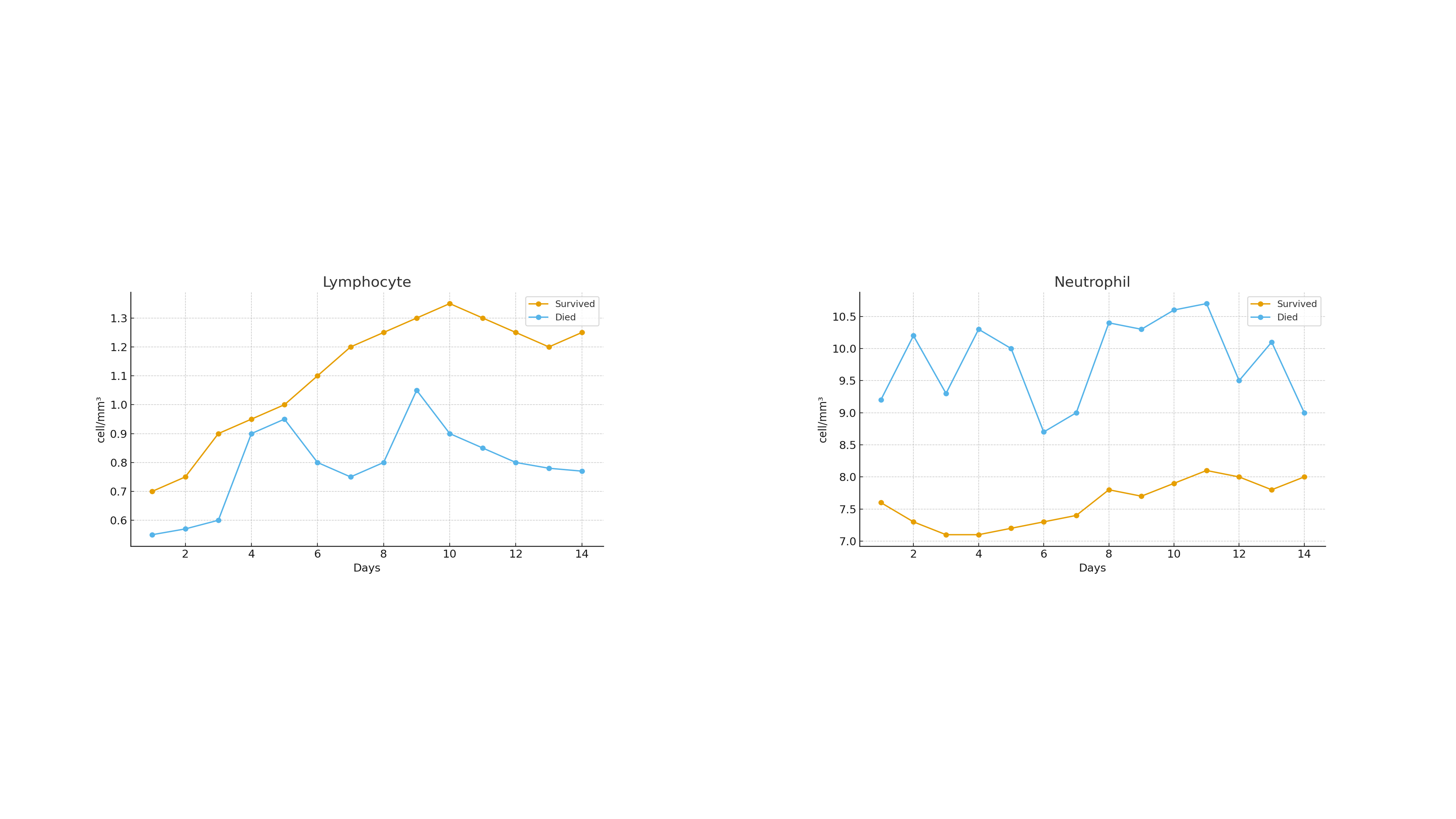

Supplement: Supplementary Figure 4 — Mean lymphocyte and neutrophil count changes in survivors and non-survivors. Days on the x-axis indicate days from anakinra initiation. Curves are displayed up to day 16 to facilitate comparison of the early treatment trajectory. The number of evaluable patients decreased over time because of discharge, death, and occasional missing measurements; therefore, later time points reflect progressively smaller denominators. [file Image4.tif]

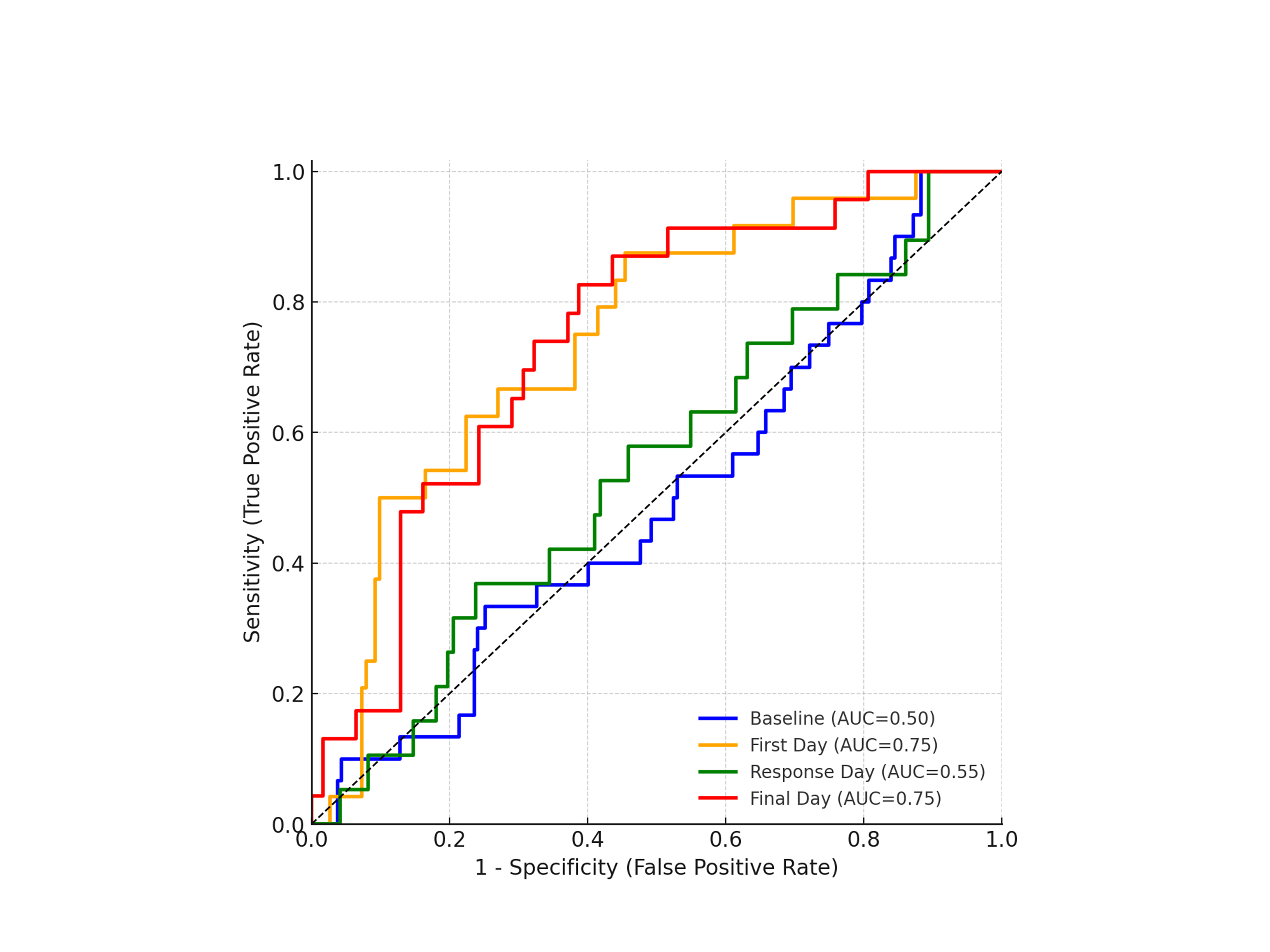

Supplement: Supplementary Figure 5 — ROC curves of HIC scores for mortality prediction. ROC curve analysis for HIC scores at different time points (baseline, day of anakinra initiation, first response day defined by ≥50% CRP reduction, and final assessment). AUC values with 95% confidence intervals (CI), p-values for the null hypothesis (AUC = 0.5), optimal cut-off values (Youden index), sensitivity, and specificity are shown. Significant discriminatory ability was observed on the day of anakinra initiation and final assessment, but not at baseline or first response day. [file Image5.tif]
