# Supplementary material for: Dynamic hyperinflammatory response assessment using HIC scores in COVID-19: application to a large series of patients receiving anakinra
Source: Front Immunol. 2026 May 22;17:1722572. doi: 10.3389/fimmu.2026.1722572 (PMC13236672; doi:10.3389/fimmu.2026.1722572)
Supplement: Supplementary Table 1 — Inflammatory parameters (mean ± SD) at baseline, day of anakinra initiation, first response day, and final assessment (discharge or death). Supplementary Table 1 presents absolute parameter values (mean ± SD) at each predefined time point. Sample size may vary across parameters and time points because of missing measurements and differences in follow-up duration. Significant differences (p < 0.05) were observed mainly at the response and final assessment stages, highlighting delayed divergence in inflammatory trajectories between survivors and non-survivors. CRP, C-reactive protein; LDH, lactate dehydrogenase; ALT, alanine aminotransferase; AST, aspartate aminotransferase. [file Table1.docx]

**Supplementary Table 1.** Inflammatory parameters (mean ± SD) at baseline, day of anakinra initiation, first response day, and final assessment (discharge or death)

|  | **Baseline** | | | **Day of anakinra initiation** | | | **First response day** | | | **Final assessment (discharge/death)** | | | | |
| --- | --- | --- | --- | --- | --- | --- | --- | --- | --- | --- | --- | --- | --- | --- |
|  | Survivors | Non-survivors | *p* | Survivors | Non-survivors | *p* | Survivors | Non-survivors | *p* | | Survivors | Non-survivors | *p* |  |
| Neutrophil (×10⁹/L) | 6.9 ± 0.31 | 6.7 ± 0.8 | 0.79 | 7.7 ± 0.3 | 8.8 ± 0.8 | 0.24 | 7.2 ± 0.27 | 9.1 ± 0.81 | 0.02 | | 6.5 ± 0.3 | 10 ± 0.7 | 0.005 |  |
| Lymphocyte (×10⁹/L) | 1.06 ± 0.22 | 0.62 ±0.57 | 0.47 | 1.1 ± 0.3 | 0.46 ± 0.9 | 0.48 | 1.2 ± 0.3 | 0.78 ± 0.9 | 0.64 | | 1.4 ± 0.06 | 1.0 ± 0.1 | 0.03 |  |
| Monocyte (×10⁹/L) | 0.44 ± 0.02 | 0.42 ±0.06 | 0.78 | 0.45 ± 0.02 | 0.40 ± 0.05 | 0.50 | 0.52 ± 0.02 | 0.53 ± 0.06 | 0.98 | | 0.59 ± 0.03 | 0.64 ± 0.09 | 0.65 |  |
| Platelet (×10⁹/L) | 237.5 ± 7.9 | 225 ±20.3 | 0.57 | 286.0 ± 9.6 | 246.2 ± 24.0 | 0.12 | 346.5 ± 10.5 | 300 ± 31.6 | 0.16 | | 332.9 ± 11 | 229.8 ± 26.4 | 0.005 |  |
| Ferritin (ng/mL) | 1249.6 ± 80.3 | 1066.7 ± 204.4 | 0.40 | 1510.7 ± 141 | 1701 ± 353.2 | 0.62 | 1270 ± 104 | 1363.9 ± 306 | 0.77 | | 773.4 ± 221.6 | 3034 ± 548.9 | 0.005 |  |
| D-dimer (ng/mL) | 2208.6 ± 302.5 | 2001.4 ± 764 | 0.80 | 1806.3 ± 210 | 2732 ± 528 | 0.10 | 1598.8 ± 179.2 | 2889.2 ± 525.7 | 0.02 | | 979.8 ± 192 | 5343 ± 464 | 0.005 |  |
| CRP (mg/L) | 114.3 ± 6 | 147.9 ± 15.8 | 0.05 | 91.4 ± 5.5 | 138.2 ± 13.9 | 0.002 | 36 ± 2.4 | 52.2 ± 7.1 | 0.03 | | 7.4 ± 4.1 | 155.8 ± 10 | 0.005 |  |
| LDH (U/L) | 395.9 ± 12.9 | 411 ± 32 | 0.66 | 403.8 ± 12 | 464.3 ± 31 | 0.07 | 357.5 ± 11.9 | 480 ± 34.9 | 0.001 | | 268.8 ± 18.8 | 601.3 ± 45.4 | 0.005 |  |
| ALT (U/L) | 44.4 ± 2.6 | 40 ± 6.6 | 0.54 | 58.5 ± 4.5 | 50.2 ± 11.9 | 0.52 | 76.4 ± 7.1 | 61.4 ± 21.1 | 0.50 | | 80.7 ± 7.7 | 124.9 ± 18.7 | 0.03 |  |
| AST (U/L) | 48.8 ± 3.3 | 43.6 ± 8 | 0.56 | 49.9 ± 3.2 | 51.8 ± 8.2 | 0.83 | 52.5 ± 5.5 | 45.9 ± 16.4 | 0.70 | | 34.7 ± 14 | 226.9 ± 35 | 0.005 |  |
| Procalcitonin (ng/mL) | 0.92 ± 0.5 | 0.81 ± 1.3 | 0.94 | 0.26 ± 0.03 | 0.57 ± 0.09 | 0.003 | 0.15 ± 0.09 | 1.2 ± 0.26 | 0.005 | | 0.2 ± 0.1 | 3.8 ± 0.4 | 0.005 |  |
| Troponin (pg/mL) | 27.5 ± 5.5 | 30.6 ± 14 | 0.84 | 67.0 ± 38 | 20.7 ± 94.8 | 0.65 | 65 ± 38.4 | 24.8 ± 111.8 | 0.73 | | 27.5 ± 7 | 127 ± 17.4 | 0.005 |  |
| Creatine kinase (U/L) | 283.4 ± 70.3 | 229 ± 202.8 | 0.80 | 214.5 ± 67.6 | 330.9 ± 175 | 0.53 | 95.6 ± 19.6 | 291 ± 57.7 | 0.002 | | 43.4 ± 20.6 | 595.3 ± 70.6 | 0.005 |  |
| Fibrinogen (mg/dL) | 612 ± 12.5 | 669.7 ± 32 | 0.09 | 611.5 ± 12.7 | 646.8 ± 32.6 | 0.31 | 547.7 ± 10.8 | 566.9 ± 32.9 | 0.58 | | 385.4 ± 10 | 532 ± 25.4 | 0.005 |  |
| Creatinine (mg/dL) | 1.2 ± 0.07 | 1.3 ± 0.1 | 0.60 | 1.1 ± 0.05 | 1.2 ± 0.1 | 0.44 | 1.1 ± 0.08 | 1.5 ± 0.2 | 0.09 | | 1.1 ± 0.08 | 1.2 ± 0.2 | 0.66 |  |

*Supplementary Table 1 presents absolute parameter values (mean ± SD) at each predefined time point. Sample size may vary across parameters and time points because of missing measurements and differences in follow-up duration. Significant differences (p < 0.05) were observed mainly at the response and final assessment stages, highlighting delayed divergence in inflammatory trajectories between survivors and non-survivors.****Abbreviations:*** *CRP, C-reactive protein; LDH, lactate dehydrogenase; ALT, alanine aminotransferase; AST, aspartate aminotransferase.*
