# Supplementary material for: Dynamic hyperinflammatory response assessment using HIC scores in COVID-19: application to a large series of patients receiving anakinra
Source: Front Immunol. 2026 May 22;17:1722572. doi: 10.3389/fimmu.2026.1722572 (PMC13236672; doi:10.3389/fimmu.2026.1722572)
Supplement: Supplementary Table 2 — Summary of inclusion criteria, glucocorticoid (GC) use, anakinra regimens, main outcomes, and mortality. A, anakinra; C, control; CRP, C-reactive protein; FiO2, fraction of inspired oxygen; PaO2, partial pressure of oxygen; suPAR, soluble urokinase plasminogen activator receptor; LDH, lactate dehydrogenase; RR, respiratory rate; IMV, invasive mechanical ventilation; WHO-CPS, World Health Organization Clinical Progression Scale. p < 0.05 indicates statistical significance where reported. [file Table2.docx]

**Supplementary Table 2.** Summary of inclusion criteria, glucocorticoid (GC) use, anakinra regimens, main outcomes, and mortality

| **Author (Year, Journal)** | **Inclusion criteria** | **GC (n)** | **Anakinra regimen** | **Main outcomes** | **Mortality (%; A vs C)** |
| --- | --- | --- | --- | --- | --- |
| Huet (2020, Lancet Rheumatol) | SpO2 ≤93% on ≥6 L/min O2 or ≥3% fall/24h | 2 | 100 mg BID ×72 h, then 100 mg QD ×7 d | IMV or death: 25% vs 73% (HR ≈0.22) | 13.5 vs 43.2 |
| Cauchois (2020, PNAS) | O2 >4 L/min; CRP ≥100 mg/L | – | 300 mg ×5 d → 200–100 mg taper | Clinical improvement; ↓O2 need; no deaths | 0 vs 10 |
| Balkhair (2020, IJID) | RR >30; SpO2 <90%; ≥6 L/min O2 | 25 | 200 mg ×3 d → 100 mg QD ≤7 d | IMV 31% vs 75% (p<0.001) | 28.8 vs 45.8 |
| Cavalli (2020, Lancet Rheumatol) | PaO2/FiO2 ≤300; CRP ≥100 mg/L; ferritin ≥900 ng/mL | 7 | 5 mg/kg BID | 21-d survival 90% vs 56% (p = 0.009) | 4.8 vs 22.5 |
| Bozzi (2021, J Allergy Clin Immunol) | Ferritin ≥1000 ng/mL; CRP ≥10 mg/dL; FiO2 ≥0.4 | 65 | 100 mg BID ×3 d → 100 mg QD ×7 d | HR 0.33 (95% CI 0.15–0.74) | 13.9 vs 35.6 |
| Pontali (2021, J Allergy Clin Immunol) | CRP or ferritin ≥3× ULN; D-dimer or LDH ≥3× ULN | 33 | 100 mg q8h (300 mg/d) with taper | Early anti-inflammatory HR 0.28 (p = 0.04) | 14.3 vs 43.2 |
| Kyriazopoulou (2021, Nat Med; SAVE-MORE) | suPAR >6 ng/mL; PaO2/FiO2 >150 | 52 | 5 mg/kg BID | 30-d mortality HR ~0.49 | 11.5 vs 22.3 |
| Kooistra (2020, Crit Care) | Fever >38.5°C ≥2 d; ferritin >1800 µg/L | 1 | 300 mg IV load → 100 mg IV q6h | No difference in IMV duration or 28-d mortality | 19 vs 17.9 |
| CORIMUNO-ANA-1 (2021, Lancet Respir Med) | O2 >3 L/min; WHO-CPS ≥5 | 7 | 200 mg BID ×3 d, taper to day 5 | No difference in primary or secondary endpoints | 22 vs 23.6 |
| Fanlo (2023, JAMA Netw Open) | SpO2 ≤93%; Pa/FiO2 ≤300; Sa/FiO2 ≤350 | 45 | 100 mg IV q6h up to 15 d | No difference in ventilation-free survival | 7.1 vs 7.4 |

**Abbreviations:** A, anakinra; C, control; CRP, C-reactive protein; FiO₂, fraction of inspired oxygen; PaO₂, partial pressure of oxygen; suPAR, soluble urokinase plasminogen activator receptor; LDH, lactate dehydrogenase; RR, respiratory rate; IMV, invasive mechanical ventilation; WHO-CPS, World Health Organization Clinical Progression Scale. p < 0.05 indicates statistical significance where reported.
